# Genetic Influences on the Covariance and Genetic Correlations in a Bivariate Twin Model: An Application to Well-Being

**DOI:** 10.1007/s10519-021-10046-y

**Published:** 2021-02-13

**Authors:** Lianne P. de Vries, Toos C. E. M. van Beijsterveldt, Hermine Maes, Lucía Colodro-Conde, Meike Bartels

**Affiliations:** 1 Department of Biological Psychology, Vrije Universiteit Amsterdam, Van der Boechorststraat 7, 1081 BT Amsterdam, The Netherlands; 2 Amsterdam Public Health Research Institute, Amsterdam University Medical Centres, Amsterdam, Netherlands; 3 Virginia Institute for Psychiatric and Behavioral Genetics, Virginia Commonwealth University, Richmond, VA USA; 4 Psychiatric Genetics, QIMR Berghofer Medical Research Institute, Brisbane, QL Australia

**Keywords:** Bivariate twin model, Bivariate heritability, Genetic correlation, Well-being, Adolescents

## Abstract

The distinction between genetic influences on the covariance (or bivariate heritability) and genetic correlations in bivariate twin models is often not well-understood or only one is reported while the results show distinctive information about the relation between traits. We applied bivariate twin models in a large sample of adolescent twins, to disentangle the association between well-being (WB) and four complex traits (optimism, anxious-depressed symptoms (AD), aggressive behaviour (AGG), and educational achievement (EA)). Optimism and AD showed respectively a strong positive and negative phenotypic correlation with WB, the negative correlation of WB and AGG is lower and the correlation with EA is nearly zero. All four traits showed a large genetic contribution to the covariance with well-being. The genetic correlations of well-being with optimism and AD are strong and smaller for AGG and EA. We used the results of the models to explain what information is retrieved based on the bivariate heritability versus the genetic correlations and the (clinical) implications.

**Supplementary Information:**

The online version contains supplementary material available at 10.1007/s10519-021-10046-y.

## Introduction

The main goal of behavior genetics research is to understand the causes of individual differences in human traits and behavior. A genetically informative design, such as the classical twin design, can be applied to decompose the observed variance of a trait into genetic and environmental sources of variation using data from reared together monozygotic (MZ) and dizygotic (DZ) twins. The difference in genetic relatedness of MZ twins (who share all genes) and DZ twins (who share on average half of their segregating genes) allows the decomposition of the observed variance in genetic and environmental sources (Boomsma et al. 2002). When investigating the sources of individual variation in a single trait, a univariate twin model is used. The heritability of the trait is estimated by dividing the genetic variance by the total variance, indicating how much of the total variance is accounted for by genetic influences. A meta-analysis of the heritability of more than 17.000 human traits showed an average heritability of 49% for all traits (Polderman et al. 2015). For most traits, the remainder of the variance is explained by non-shared environmental components and measurement error, whereas the influences from shared environmental or dominant genetic effects are usually absent or small.

By extending the univariate model, two or more traits can be included in twin models to understand the causes of covariation between traits (Eaves and Gale 1974; Martin and Eaves 1977). In a bivariate (two traits) or multivariate (multiple traits) model, the covariation between two or more traits is decomposed in genetic and environmental components, in addition to the trait specific variance decomposition. The covariance decomposition answers the question how much of the phenotypic correlation between the traits is accounted for by genetic and environmental factors. The proportion of covariance explained by genetic factors is called bivariate heritability.

Additionally, in a bi- or multivariate model, the genetic and environmental correlations between traits can be computed. The genetic correlation reflects the extent to which the genetic factors underlying one trait overlap with the genetic factors that influence the other trait in the model. Similarly, the environmental correlation reflects the overlap of the environmental factors underlying the traits. A genetic (or environmental) correlation of 1 indicates a perfect overlap of the genetic (or environmental) factors, indicating that the factors that influence both traits are identical. In contrast, a correlation of 0 indicates no overlap and thus independent genetic (or environmental) factors for both traits.

For example, applying a bivariate model to well-being and depressive symptoms, Baselmans et al. (2018) found that additive genetic effects explained 46% of the covariance between these traits in adults. This bivariate heritability means that 46% of the phenotypic correlation between well-being and depressive symptoms is accounted for by genetic factors, whereas the remainder is explained by unique environmental factors. In addition, the modelling resulted in strong genetic (−0.60) and environmental (−0.48) correlations. The genetic correlation of −0.60 indicates that more than half of the genetic factors influencing well-being also have an influence on depression. The negative signs indicate that the effect of the genetic factors on the traits is in the opposite direction, so those genetic factors that increase well-being, also decrease depressive symptoms and vice versa. The same applies to the negative environmental correlation. Around half of the environmental factors influencing well-being are shared with and have an opposite effect on depressive symptoms.

### Goal of the present study

In behavior genetics studies, often the distinction between the results of the covariance decomposition and the genetic/environmental correlation is either not well-understood or only one of the two is reported while the results show distinctive information about the covariance and overlap between traits. For example, while the influence of genetic factors on the phenotypic correlation can be rather substantial, the genetic correlation can be low, indicating that although genes play an important role in the observed overlap between traits, most of the genetic influences are trait specific. Or the opposite can be observed, that is while there is only a small influence of genetic factors on the covariance, the genetic correlation is substantial. This indicates that the sets of genes that influence each trait are shared to a large extent, but that genes are actually not that important in understanding the association between two traits.

Therefore, in this paper we will explain the difference between the results of the covariance decomposition and genetic and environmental correlations in greater depth. Given our longstanding interest, we will apply the bivariate model to well-being and four other traits known to have a different phenotypic association with well-being in a large sample of adolescent twins. The four traits included are optimism, symptoms of anxiety or depression, aggressive behavior, and educational achievement. We chose these traits based on previous work (summarized below), since we expect different associations of the traits with well-being (i.e. different phenotypic correlations, bivariate heritability estimates and genetic and environmental correlations). These differences (e.g. high/low bivariate heritability and high/low genetic correlation) have different (clinical) implications and will help clarify the difference between both sets of results of the bivariate twin model.

Well-being is characterized by high levels of positive affect, low levels of negative affect, and a positive subjective evaluation of life satisfaction (Diener et al. 2018). As our well-being measure we include satisfaction with life, i.e. the subjective evaluation of life. Using slightly different inclusion criteria, two meta-analyses summarized all studies applying the twin model to well-being and found a meta-analytic heritability of 36% (CI 34–38%) and 40% (CI 38–43%) for well-being based on all measures (Nes and Røysamb 2015; Bartels 2015) and 32% (CI 29–35%) for satisfaction with life (Bartels 2015).

Optimism can be defined as the tendency to expect positive outcomes in the future in any situation and is, like well-being, related to physical and mental health (Scheier and Carver 1992, 1993; Rasmussen et al. 2009). A large meta-analysis showed strong phenotypic correlations between optimism and the different aspects of well-being in large samples, e.g. the correlations with life satisfaction (N = 19,831), quality of life (N = 2824) and happiness (N = 5470) were 0.55, 0.53, and 0.36 respectively (Alarcon et al. 2013). Furthermore, like well-being, individual differences in optimism have been found to be accounted for by genetic influences (around 30%) and non-shared environmental influences (Plomin et al. 1992; Caprara et al. 2009; Yuh et al. 2010; Mosing et al. 2010; Bates 2015; Mavioğlu et al. 2015).Therefore, we expect a genetic contribution to the strong correlation between well-being and optimism and an overlap in the genetic and environmental factors underlying both traits, resulting in strong genetic and environmental correlations.

Anxious-depressed symptoms refer to the number of anxiety and depression related symptoms experienced. The negative moderate phenotypic association between well-being and these internalizing symptoms is well-established. In a recent study, Baselmans et al. (2018) investigated the contribution of genetic and environmental factors to the relation between well-being and depressive symptoms across the lifespan. In adolescents, a phenotypic correlation of -0.47 in adolescents, a high bivariate heritability (68%) and strong genetic (−0.59) and environmental (−0.32) correlations were found. Given the overlap in sample used for this study we expect similar results, but we included it as comparison to the other bivariate results.

Aggressive behavior can be defined as any threatening, upset, verbally or physically violent behavior (Achenbach and Rescorla 2001). Meta-analytic heritability estimates of 48–65% have been found for aggression (Miles and Carey 1997; Burt 2009). Between well-being and aggression, a moderately negative correlation (around −0.2) is reported, indicating that happier adolescents display less violent and aggressive behavior (Valois et al. 2001; Macdonald et al. 2005; Buelga et al. 2008). Furthermore, large genetic contributions (72% and 90% in adolescent males and females respectively) to the covariance and moderate genetic correlations (−0.38 and −0.39) between well-being and aggressive behavior have been found (Bartels et al. 2013). In our sample, we expect a similar genetic contribution to the covariance and overlap in genetic and environmental factors.

Lastly, we included educational achievement or performance, as the literature describes only a slightly positive association with well-being in adolescents (e.g. Gilman and Huebner 2006; Verkuyten and Thijs 2002). In children and adolescents, differences in educational achievement are highly heritable in primary and secondary education (around 60%) (Bartels et al. 2002; Krapohl et al. 2014; de Zeeuw et al. 2015). Despite the high heritability of educational achievement, we expect a small genetic contribution to the association between well-being and educational achievement, with most of the genetic influences being trait specific. Furthermore, the small phenotypic correlation suggests low genetic and environmental correlations with well-being.

## Method

### Participants

Participants are registered at the Netherlands Twin Register (NTR), established by the Department of Biological Psychology, Vrije Universiteit Amsterdam (Van Beijsterveldt et al. 2013; Ligthart et al. 2019). The NTR sample is a population-wide, non-clinical sample, including children, adolescents, and adults. Young twins are registered in the Young Netherlands Twin Register (YNTR) at birth by their parents with the help of a commercial ‘birth felicitation’ service and the support of the Dutch Society of Parents of Multiples (Nederlandse Vereniging van Ouders van Meerlingen: NVOM; https://www.nvom.nl) (Van Beijsterveldt et al. 2013; Ligthart et al. 2019). In childhood, the parents were asked to complete questionnaires about their children. From the age of 14, the twins and their siblings were invited, after parental consent, to complete questionnaires themselves. The current study used data of the Dutch Health and Behavior Questionnaire (DHBQ), a self-report instrument which assesses health, lifestyle, and behaviour when twins are 14 and 16 years old (Bartels et al. 2011; Van Beijsterveldt et al. 2013).

We used DHBQ data of 14-year-old same-sex twins. To increase the sample size, for twins without data in the 14-year sample, we included data collected at age 16, if available. The final sample included data of 9648 twins with an average age of 15.18 years old (SD = 1.13), 42.3% of the participants were male. The sample included 5242 MZ twins and 4442 DZ twins, from complete or incomplete same sex twin pairs. For the bivariate models, subsets of the sample were used with data on well-being and optimism (N = 8697; MZ = 4696, DZ = 4001)), well-being and anxious/depressed symptoms (N = 8363; MZ = 4541, DZ = 3822), well-being and aggressive behavior (N = 8351; MZ = 4536, DZ = 3816) and well-being and educational achievement (N = 8076; MZ = 4383, DZ = 3693).

Zygosity for same-sex twin pairs was determined by DNA analysis (27%) or by previously collected parental report on physical similarity and the frequency of confusion of the twins by parents, other family members and strangers (Willemsen et al. 2013). Agreement between the replies to the longitudinal questionnaire and DNA determined zygosity was around 93% (Rietveld et al. 2000)*.*

## Material

### Well-being

Well-being was assessed using the Satisfaction with Life scale (Diener et al. 1985). The Satisfaction with Life scale has five items to report life satisfaction on a 7-point Likert scale, ranging from 1 = *strongly disagree* to 7 = *strongly agree*. An example item is ‘*In most ways my life is close to ideal*’. As in all following scales, the total score was only computed when all items were completed, i.e. when participants had no missing data. The Satisfaction with Life scale shows high internal consistency and high temporal reliability (2-month test–retest: 0.82, and coefficient alpha was 0.87) (Diener et al. 1985). The internal consistency of the SWLS in our sample is similar, with a Cronbach’s alpha of 0.86.

### Optimism

Optimism was assessed with the revised Life Orientation Test (LOT-R) (Scheier et al. 1994). The scale consists of 10 items, three for optimism, three for pessimism, and four filler items. Participants had to rate their agreement with the items on a 5-point Likert scale (1 = *strongly disagree*, 5 = *strongly agree*). As Mavioğlu et al. (2015) concluded that the LOT-R should be considered a bi-dimensional scale with two correlated constructs of optimism and pessimism, we summed the scores on the three optimism items to create an optimism score instead of using all six optimism and (reverse coded) pessimism items. An example item is “*In uncertain times, I usually expect the best*”. The LOT-R has a high internal consistency (Cronbach’s alpha = 0.78) and temporal reliability (4 month test–retest: 0.68) (Scheier et al. 1994).

### Anxious-depressed symptoms

Symptoms of anxiety and depression were assessed with the Anxious-Depressed subscale of the ASEBA Youth Self Report (Achenbach and Rescorla 2001). This subscale consists of 13 items and adolescents have to rate the occurrence of the behavior, now or within the past 6 months, on a 3-point scale (0 if the problem item is not true, 1 if it is sometimes true, and 2 if it is very true or often true). An example item is “*I am unhappy, sad, or depressed*”. The anxious-depressed score was computed by summing the items. The internal consistency (Cronbach’s alpha = 0.87) and temporal reliability (1 week test–retest: 0.88) of the anxious-depressed scale are both high (Achenbach and Rescorla 2003).

### Aggressive behavior

Aggressive behavior was assessed with the Aggressive Behavior subscale of the ASEBA Youth Self Report (Achenbach and Rescorla 2001). This subscale consists of 17 items and adolescents have to rate the occurrence of certain behavior, now or within the past 6 months, on a 3-point scale, ranging from 0 (not true) to 2 (very true). An example item is “*I get in many fights*”. The aggressive behavior score was computed by summing the items. The internal consistency (Cronbach’s alpha = 0.87) and temporal reliability (1 week test–retest: 0.83) of the aggressive behavior scale are both high (Achenbach and Rescorla 2003).

### Educational achievement

For educational achievement, we used the Dutch Cito-elementary test score. The Cito-test is a standardized test for educational achievement in the Netherlands. Children take the test in the final grade of elementary school (around 11 or 12 years old) (see Eindtoets Basisonderwijs; www.cito.nl). The Cito-test consists of four parts, including items on language, mathematics, study skills, and world orientation. Combining the scores on the four tests results in a standardized Cito-score, ranging from 500 to 550. The Cito-test results are used in the primary school’s advice on the most appropriate level of secondary education and reflects educational achievement. In the DHBQ, adolescents were asked whether they took the Cito-test and if so, to report their Cito score.

### Statistical analysis

To decompose the variances and covariance in genetic and environmental components and to estimate genetic and environmental correlations between well-being and each of the four different traits, we conducted bivariate twin models using data of same sex MZ and DZ twins. Twin models use the different degree of genetic relatedness between monozygotic (share 100% of their genes) and dizygotic twins (share on average 50% of their genes) to decompose the variance and covariance in genetic and environmental components. In bivariate models, the within-twin cross-trait covariances/correlations show whether there are common etiological influences in the traits; importantly, the cross-twin cross-trait covariances/correlations give information about whether familial (genetic or environmental) common etiological influences contribute to the covariation.

The genetic variance and covariance components are additive genetic variance (A) and non-additive genetic variance (D). Whereas A represents the additive variance explained by summing the effects of all alleles that influence the phenotype, D arises due to interactions between alleles at the same locus (dominance) or between alleles at different loci (epistasis) and cannot be explained by a linear model. The environmental variance and covariance consist of a common environmental variance component (C) (variance shared by family members) and a non-shared environmental component (E) (unique for an individual). The effects of C and D cannot be estimated in the same model unless an extended family design is applied. Therefore, a choice for an ACE or ADE model was made based on the ratio of the cross-twin cross-trait correlations. If the MZ correlations are smaller than twice the DZ correlations, common environment (C) effects are expected and an ACE model is used. If the MZ correlations are larger than twice the DZ correlations, dominant genetic (D) effects are expected and an ADE model is appropriate (Neale and Maes 2004). We used the variance component approach, which has better statistical properties (see Verhulst et al. 2019).

First, phenotypic correlations, twin correlations, and cross-twin cross-trait correlations were estimated using a saturated model in OpenMx in R (Boker et al. 2011). Next, using a bivariate ACE or ADE model, we estimated genetic and environmental contributions to variance of the traits and to the bivariate phenotypic covariance,—that is, how much of the phenotypic correlation between the traits is accounted for by genetic and (shared and unique) environmental factors. Additionally, we obtained the genetic and environmental correlations between the traits. The genetic correlation is computed by dividing the genetic covariance by the square root of the product of the genetic variances of the two variables $\left( {{\text{r}}_{{\text{g}}} = {{{\text{VA}}12} \mathord{\left/ {\vphantom {{{\text{VA}}12} {\sqrt {{\text{VA}}11{\text{*VA}}22} }}} \right. \kern-\nulldelimiterspace} {\sqrt {{\text{VA}}11{\text{*VA}}22} }}} \right)$. Similarly, the (shared or unique) environmental correlation is computed by dividing the (shared or unique) environmental covariance by the square root of the product of the (shared or unique) environmental variances of the two variables $\left( {{\text{r}}_{{\text{e}}} = {{{\text{VE}}12} \mathord{\left/ {\vphantom {{{\text{VE}}12} {\sqrt {{\text{VE}}11{\text{*VE}}22} }}} \right. \kern-\nulldelimiterspace} {\sqrt {{\text{VE}}11{\text{*VE}}22} }}} \right)$. These correlations reflect the overlap between genetic and environmental factors underlying the traits. The scripts we used for the bivariate saturated and ACE/ADE model can be found in the supplementary information.

First, we tested the contribution of the C or D component to the total variance and covariances between traits. However, if there are sex differences in the factors contributing to the variation, dropping 6 parameters might result in a significant deterioration of the fit, even though the contribution of C/D is small. Therefore, based on the twin correlations and the full model, we tested the contribution of C/D separately for males and females if necessary.

Next, we tested for quantitative sex differences in the variance components (A, possibly C/D and E) by constraining the variance estimates of both phenotypes and the covariance between the phenotypes of females and males to be equal. We did not test for qualitative sex differences, as modelling sex specific genes in multivariate models has inherent limitations (Neale et al. 2006) and no qualitative sex effects in well-being are expected based on the literature (Stubbe et al. 2005) or are found in univariate models of the traits.

The fit of the different models and nested models (both for the saturated as well as the genetic models) was compared by means of the log-likelihood ratio test (LRT). The difference in minus two times the log-likelihood (−2LL) between two nested models has a χ^2^ distribution with the degrees of freedom (df) equalling the difference in df between the two models. If a p value from the χ^2^ -test is significant (threshold for the genetic models p < 0.01 due to multiple testing), the fit of the constrained model is significantly worse than the fit of the more complex model. For the best fitting model, 95% confidence intervals were estimated for the parameters.

## Results

Table 1 contains the means and standard deviations for all the measures. Based on the saturated model results, there were significant mean differences between females and males for all measures. Males scored higher on well-being, optimism, aggressive behavior, and educational achievement, whereas females scored higher on anxious-depressed symptoms (all: p < 0.001). All phenotypic correlations between well-being and the measures were significant and are reported in Table 2 separately for females and males. The correlations with optimism and anxious-depressed symptoms were moderately positive and negative respectively. The negative correlation of well-being with aggressive behavior was lower and the positive correlation with educational achievement was close to zero.

**Table 1:** Descriptives of the measures, separately for females and males

	Females				Males			
	N	M	SD	Range	N	M	SD	Range
Well-being	4687	27.21	5.34	5–35	3338	27.84	4.95	5–35
Optimism	3598	10.09	1.78	3–15	2475	10.64	1.74	3–15
Anxious-depressed symptoms	4493	5.04	3.85	0–23	2849	3.23	2.89	0–26
Aggressive behavior	4497	4.68	3.49	0–27	3203	4.97	3.87	0–34
Educational achievement	2705	537.78	8.62	501–550	1927	539.52	8.07	503–550

Sex differences are significant for all measures, p < 0.001

**Table 2:** Phenotypic correlations with well-being (Satisfaction with Life), with 95% confidence intervals

	Females	Males
Optimism	0.403 (0.374, .432)	0.334 (.295, .372)
Anxious-depressed symptoms	−0.460 (−0.484, −0.435)	−0.337 (−0.371, −0.301)
Aggressive behavior	−0.238 (−0.268, −0.209)	−0.096 (−0.160, −0.031)
Educational achievement	0.060 (0.018, 0.101)	0.057 (0.007, 0.106)

The twin correlations (see Table 3) showed that for all measures part of the variance is explained by additive genetic influences, as the MZ correlation was consistently higher than the DZ correlation. The cross-twin cross-trait correlations are higher for MZ than for DZ twins as well, indicating an influence of genetic effects on the association between well-being and the other measures. As there was no evidence for dominant genetic effects, we continued with ACE models.

**Table 3:** Cross-twin cross-trait correlations

	MZf/DZf		MZm/DZm	
	WB	Opt	WB	Opt
WB	0.409/0.286		0.374/0.198	
Opt	0.249/0.163	0.274/0.247	0.139/0.121	0.261/0.111

	WB	Anx-Dep	WB	Anx-Dep
WB	0.402/0.280	–	0.394/0.224	
Anx-Dep	−0.264/−0.160	0.395/0.214	−0.301/−0.226	0.527/0.378

	WB	Aggr	WB	Aggr
WB	0.412/0.480		0.379/0.187	
Aggr	−0.167/−0.106	0.532/0.252	−0.114/−0.109	0.426/0.223

	WB	Educ	WB	Educ
WB	0.411/0.278		0.378/0.189	
Educ	0.038/0.020	0.799/0.378	0.070/−0.002	0.812/0.478

*WB* well-being, *Opt* optimism, *Anx-Dep* anxious-depressed symptoms, *Aggr* aggressive behavior, *Educ* educational achievement

The model fitting results showed that there were quantitative sex differences in all bivariate models. The best fitting model for all phenotypes using an alpha of 0.01 was an AE model for both females and males (see Table 4). In supplementary Table S1, and S2 the standardized and unstandardized variance estimates of males and females in the full ACE models can be found. In Table S3 the genetic and environmental correlations of the full ACE models can be found.

**Table 4:** Model fitting results of the bivariate models with well-being

Optimism	Base	Comparison	ep	-2LL	df	AIC	ΔLL	Δdf	p
1	ACE		22	71,574.8	14,019	43,536.8			
2	ACE	**AE with sex diff**	16	71,591.5	14,025	43,541.5	16.7	6	**0.0105**
3	AE	No sex diff	10	71,622.4	14,031	43,560.4	30.9	6	< .0001

Anx-Dep symptoms	Base	Comparison	ep	-2LL	df	AIC	ΔLL	Δdf	p
1	ACE		22	85,432.1	15,241	54,950.1			
2	ACE	**AE with sex diff**	16	85,440.7	15,247	54,946.7	8.6	6	**0.1954**
3	AE	No sex diff	10	85,701.5	15,253	55,195.5	260.8	6	< .0001

Aggressive behavior	Base	Comparison	ep	-2LL	df	AIC	ΔLL	Δdf	p
1	ACE		22	88,937.8	15,594	57,749.8			
2	ACE	**AE with sex diff**	16	88,947.0	15,600	57,747.0	9.2	6	**0.1638**
3	AE	No sex diff	10	89,027.7	15,606	57,815.7	80.7	6	< .0001

Educational achievement	Base	Comparison	ep	-2LL	df	AIC	ΔLL	Δdf	p
1	ACE		22	79,796.7	12,554	54,688.7			
2	ACE	**AE with sex diff**	16	79,805.8	12,560	54,685.8	9.1	6	**0.1694**
3	AE	No sex diff	10	79,837.6	12,566	54,705.6	31.8	6	< .0001

*Base* baseline model, *ep* estimated parameters, − *2LL* = minus two times the log-likelihood, *df* degrees of freedom, *AIC* akaike information criterion, *p* = p-value, *Anx-Dep symptoms* anxious-depressed symptoms; best-fitting model in bold letters

**Fig. 1:**
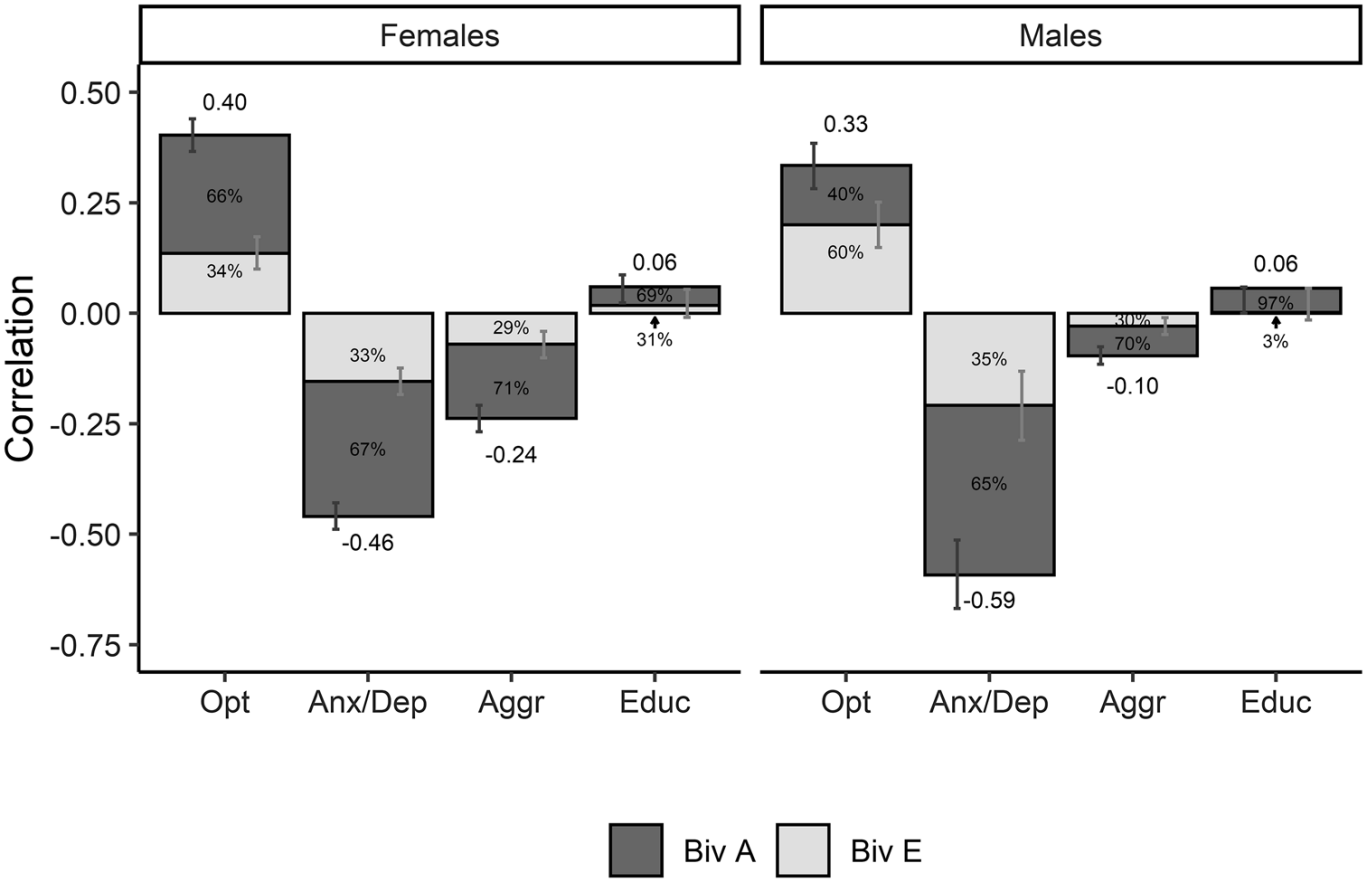
Phenotypic correlations between well-being and the traits optimism, anxious-depressed symptoms, aggressive behavior and educational achievement with the proportions that are accounted for by additive genetic (Biv A) and non-shared environmental influences (Biv E). The error bars indicate the 95% confidence intervals

Table 5 shows the standardized bivariate genetic and environmental estimates for the variance and covariance of the four best fitting models (the unstandardized estimates can be found in supplementary Table S4). The phenotypic correlations between well-being and the four other measures and the proportions that were accounted for by genetic (i.e. the bivariate heritability), and non-shared environmental influences are presented in Fig. 1. Genetic factors contributed to the covariance with well-being in all four traits to a different extent.

**Table 5:** The bivariate estimates (with 95% confidence intervals) of the variance and covariance of well-being and the four other phenotypes of the four best fitting bivariate models

	A		E	
	WB	Opt	WB	Opt
Females				
WB	0.444 (0.401, 0.484)		0.556 (0.516, 0.599)	
Opt	0.663 (0.570, 0.752)	0.311 (0.256, 0.363)	0.337 (0.248, 0.430)	0.689 (0.637, 0.744)
Males				
WB	0.344 (0.284, 0.400)		0.656 (0.600, 0.716)	
Opt	0.403 (0.246, 0.554)	0.244 (0.171, 0.314)	0.597 (0.446, 0.754)	0.756 (0.686, 0.829)

	A		E	
	WB	Anx-Dep	WB	Anx-Dep
Females				
WB	0.443 (0.400, 0.483)		0.557 (0.517, 0.600)	
Anx-Dep	0.666 (0.599, 0.730)	0.444 (0.400, 0.485)	0.334 (0.270, 0.401)	0.556 (0.515, 0.600)
Males				
WB	0.364 (0.287, 0.402)		0.654 (0.598, 0.713)	
Anx-Dep	0.649 (0.515, 0.778)	0.394 (0.330, 0.452)	0.351 (0.222, 0.485)	0.606 (0.548, 0.670)

	A		E	
	WB	Aggr	WB	Aggr
Females				
WB	0.444 (0.401, 0.484)		0.556 (0.516, 0.599)	
Aggr	0.706 (0.578, 0.829)	0.495 (0.451, 0.536)	0.294 (0.171, 0.422)	0.505 (0.464, 0.549)
Males				
WB	0.344 (0.284, 0.400)		0.656 (0.600, 0.716)	
Aggr	0.699 (0.496, 0.900)	0.459 (0.406, 0.509)	0.301 (0.100, 0.504)	0.541 (0.491, 0.594)

	A		E	
	WB	Educ	WB	Educ
Females				
WB	0.444 (0.402, 0.485)		0.556 (0.510, 0.598)	
Educ	0.693 (0.100, 1.15)	0.804 (0.778, 0.826)	0.307 (-0.15, 0.900)	0.196 (0.174, 0.222)
Males				
WB	0.346 (0.286, 0.402)		0.654 (0.598, 0.714)	
Educ	0.966 (NA, NA)	0.801 (0.769, 0.828)	0.034 (NA, NA)	0.199 (0.172, 0.231)

*WB* well-being, *Opt* optimism, *Anx-Dep* anxious-depressed symptoms, *Aggr* aggressive behavior, *Educ* educational achievement

**Fig. 2:**
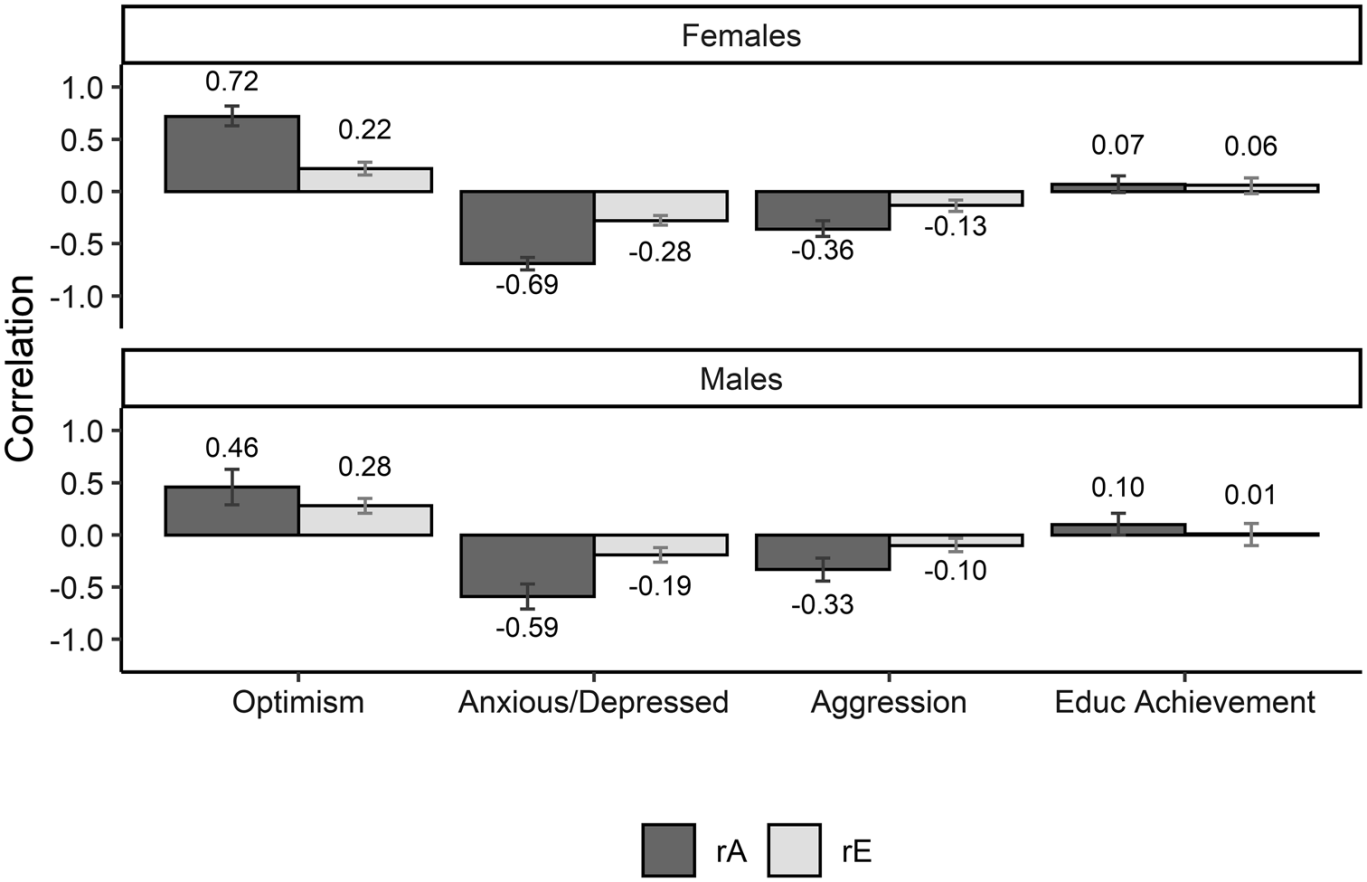
The genetic (rA) and non-shared environmental correlations (rE) between well-being and the other traits, optimism, anxious-depressed symptoms, aggressive behavior and educational achievement. The error bars indicate the 95% confidence intervals

In Fig. 2 (and supplementary Table S5) the genetic and non-shared environmental correlations between well-being and the four measures can be found. The genetic correlations ranged from 0.07 to 0.72 and environmental correlations ranged from 0.01 to 0.28 across the traits, indicating variation in the genetic and environmental overlap of the traits with well-being.

### Optimism

The phenotypic correlation between optimism and well-being (r_females_ = 0.40 and r_males_ = 0.33) was explained by genetic influences for respectively 66% and 40% in females and males, whereas the rest was explained by non-shared environmental influences.

The genetic correlations between well-being and optimism were relatively strong in females (rg_females=_ 0.72) and lower in males (rg_males_ = 0.47), indicating that the genetic factors underlying both traits overlap to a significant extent and to a greater extent in females than in males. In addition, the non-shared environmental correlations were moderate, with 0.22 and 0.28 for females and males, respectively, indicating an overlap in environmental factors as well.

### Anxious-depressed

The phenotypic correlation between well-being and anxious-depressed symptoms (r_females_ = −0.46 and r_males_ = −0.34) was primarily influenced by genetic influences (67% and 65% for females and males respectively), whereas the rest was explained by non-shared influences.

The genetic correlation between well-being and anxious-depressed symptoms was negative and strong in both sexes (rg_females_ = −0.69, rg_males_ = −0.59) as well, indicating that there were largely overlapping genetic influences on the phenotypes. The non-shared environmental correlations were −0.28 and −0.19 for females and males respectively. The negative correlations indicated that the effects of the influences are in opposite directions.

### Aggressive behavior

For well-being and aggressive behavior, the phenotypic correlation (r_females_ = −0.24 and r_males_ = −0.10) was mainly explained by genetic influences (71% and 70% respectively), whereas the rest was explained by non-shared environmental influences.

The genetic correlation between well-being and aggressive behavior was moderately negative (rg_females_ = −0.36 and rg_males_ = −0.33). Overlapping genetic factors influence both well-being and aggressive behavior, but the effects of the genetic factors on the traits were opposite. The non-shared environmental correlations were −0.13 in females and −0.10 in males, indicating some overlap in the environmental factors underlying the traits as well, and they also went in the opposite direction.

### Educational achievement

Lastly, the small phenotypic correlation between well-being and educational achievement (r = 0.06 for both sexes) was made up of genetic (69% and 97%) and non-shared environmental (31% and 3%) influences in females and males respectively. However, there are large confidence intervals, as can be seen in Fig. 1.

The genetic and environmental correlations between well-being and educational achievement were close to zero (< 0.10) in both sexes, with zero in the confidence intervals, indicating no significant overlapping genetic and environmental influences on well-being and educational achievement.

## Discussion

The four bivariate twin models fitted to data of adolescent females and males using well-being (WB; satisfaction with life) and four other complex traits, namely optimism, anxious-depressed symptoms, aggressive behavior, and educational achievement resulted in variability in the phenotypic correlations, bivariate heritability, and genetic and environmental correlations. Bivariate models like the ones that we applied in this paper provide insight into the underlying source of overlap between two traits and provide an indication of how strong the genetic link is between two phenotypes. If interested in exploring the relationship between all these traits taken together, other models (e.g. multivariate models) that were not the scope of this paper have to be fitted.

The bivariate heritability indicates the proportion of the correlation between the phenotypes that is accounted for by additive genetic influences. While the phenotypic correlation between WB and the four phenotypes varied from moderate (r about 0.4 for WB with optimism and anxious-depressed symptoms) to small (around 0.2 for WB with aggressive behavior and 0.05 with educational achievement), a significant part of all these varying phenotypic correlations was accounted for by genetic factors. For optimism, anxious-depressed symptoms and aggressive behavior the bivariate heritability with well-being ranged between 0.40 and 0.70, indicating that around half to more than half of the overlap between well-being and these phenotypes can be explained by genetic factors. For educational achievement, the estimates for the bivariate heritability are 69% and even 97% in females and males respectively, indicating that almost all covariance between educational achievement and well-being was explained by genetic factors. However, as the phenotypic correlation between well-being and educational achievement is really small and the confidence intervals around the bivariate heritability estimate were large, this estimate may not be relevant and must be interpreted with care.

The genetic correlation reflects a measure of overlap in the genetic factors underlying the traits and is different than the bivariate heritability. For example, if the bivariate heritability is large and the genetic correlation is low, genes do play a substantial role in the observed overlap between traits, but most of the genetic influences are trait specific. In our examples, the bivariate heritability between well-being and anxious-depressed symptoms and well-being and aggressive behavior was similar, whereas the genetic correlation between well-being and anxious-depressed symptoms was higher than between well-being and aggressive behavior. This indicated that genes play a role in the overlap between WB and the traits to a similar extent, whereas the biological mechanisms underlying well-being and anxious-depressed symptoms are more similar than those underlying well-being and aggressive behavior. The genetic correlation between well-being and optimism was relatively strong as well, indicating that the biological mechanism underlying the positive traits of well-being and optimism is similar as well. The genetic factors underlying well-being and educational achievement overlapped only a little or not at all, as indicated by a genetic correlation lower than 0.1 suggesting that the biological mechanisms underlying well-being and educational achievement are almost completely separate.

A similar pattern can be observed in the unique environmental influences on the covariance between the traits and the unique environmental correlations between well-being and the four traits. Although the phenotypic correlations between well-being and the other traits vary, roughly a similar proportion is accounted for by environmental factors. Additionally, as indicated by the environmental correlations, more environmental factors underlying well-being and both optimism and anxious-depressed symptoms (r_e_ ~ 0.30) overlapped compared to aggressive behavior (r_e_ ~ 0.10). For educational achievement, none of the environmental factors overlapped, indicated by an environmental correlation of zero.

For every bivariate model, the best fitting model included quantitative sex differences, i.e. the (bivariate) heritability for males and females differed. Sex differences in the genetic architecture of traits might be quantitative (either scalar or non-scalar) or qualitative. As mentioned before, the literature and our data indicated no qualitative sex differences, therefore we did not test these sex differences. An alternative model, to the one used in the paper, to test non-scalar quantitative sex differences is the scalar sex limitation model. In this model, the total variance in males and females is allowed to differ by a factor k (i.e. variance_female_ = k * variance_male_), whereas the heritability is constrained to be equal. We compared the fit of both quantitative sex limitation models. Only for the bivariate model of well-being and educational achievement, the scalar sex limitation model resulted in a better fit than the non-scalar bivariate model (Δ-2LL = 13.9, Δdf = 8, p = 0.085). This indicates scaled sex differences in the unstandardized variance components, but no sex differences in the standardized variance components (i.e. the heritability). However, the difference between the standardized variance components of this scalar AE model and the reported non-scalar AE model were small.

### Added value of bivariate twin models

An advantage of the bivariate or multivariate models is the increase in power compared to the univariate twin model (Schmitz et al. 1998). As in all models, the size of the sample and the ratio of MZ and DZ twin pairs is important for sufficient power. However, often in univariate models, there is not enough power to discriminate between genetic and shared environmental variance components for a single trait. In bivariate models, the within twin pair covariance and the cross-trait and within-person covariance provide unique additional information, leading to more powerful models to estimate both the heritability of the single traits and the bivariate heritability. However, for bivariate models to increase the power, there has to be a significant phenotypic correlation between the traits (Schmitz et al. 1998). If there is only a small phenotypic correlation between the traits, like in our well-being and educational achievement example, the models will still be underpowered. This is also indicated by the large confidence interval around the bivariate heritability in the educational achievement example.

Furthermore, the value of bivariate twin models also rests in the genetic correlations, which can be compared and provide a reference for those based on molecular genetic data. Molecular genetic correlations can be estimated using summary statistics of large Genome Wide Association Studies (GWAS) and LD score regression (Bulik-Sullivan et al. 2015). Comparing the results of our twin model and recent GWASs, the genetic correlations are very similar. For anxious-depressed symptoms and well-being, the genetic correlation in our twin study was -0.69 in females and -0.59 in males, whereas the molecular genetic correlation between well-being and depressive symptoms (without taking sex differences into account) is estimated around −0.70 (Okbay et al. 2016; Baselmans et al. 2019). For aggressive behavior and well-being, the genetic correlation in our study was −0.36 in females and −0.33 in males. The molecular genetic correlation based on GWAS summary statistics is almost the same and estimated at −0.39 (Ip et al. 2019). Finally, for educational achievement the genetic correlation in our study was non-significant and estimated at 0.07 in females and 0.11 in males. The molecular genetic correlation is low as well and slightly negative, with −−0.15 (Baselmans et al. 2019). Therefore, using far fewer participants and other (frequently less expensive) resources, the genetic correlations based on twin models provide reliable estimates about the overlap in genes between complex traits. Therefore, twin studies and the large longitudinal phenotypically rich twin registries all around the world provide a valuable resource for a first indication of genetic overlap between traits or stability of genetic influences over time.

Besides investigating the overlap between two or more traits, different forms of bivariate or multivariate twin models exist. Examples of other powerful bivariate models are a multiple rater twin model or a multiple measurement model of one trait (or more traits) over time (Hewitt et al. 1992; Bartels et al. 2007). In a multiple rater model, multiple informants, such as the parents and/or teacher rate a child on specific traits. Based on common (co)variance, the sources of overlap between two traits can be estimated more reliably as the measurement error decreases (e.g. see (Bartels et al. 2003) for a bivariate model including two traits and two raters). Similarly, bivariate or multivariate twin models can be applied to longitudinal data including data of multiple time points to give insight into the development of traits over time. In such models, the genetic and environmental correlations reflect the overlap of genetic and environmental factors over time and thus the underlying stability of the trait (e.g. Kan et al. 2013; Nivard et al. 2015; Baselmans et al. 2018). For example, the bivariate heritability and genetic correlations between the measurements of anxiety and depression symptoms across the life span indicated that the stability in anxiety or depression symptoms is mainly due to genetic effects, with the importance of environmental effects increasing with age (Nivard et al. 2015).

### Implications

The results of bivariate twin modelling in different forms can lead to information about the overlap between traits and about the development over the life span. This information can be used to inform the development of prevention and intervention programs for psychopathology. For example, returning to our well-being application, prevention of psychopathology and increasing well-being in adolescence is of great importance. The genetic correlations between well-being and anxious-depressed symptoms in our adolescent sample indicated a high overlap in the biological mechanisms underlying the traits. With a high genetic correlation, a genetic liability for lower well-being can be indicative for a genetic liability for higher psychopathology. People at risk for psychopathology can be identified based on their well-being before any symptoms start to develop. Similarly, a high environmental correlation indicates that the same environmental influences have both an effect on well-being and psychopathology. Although well-being and symptoms of anxious-depressed are different and do not lie on the same continuum (e.g. Howell et al. 2007; Ryff et al. 2006), the results do suggest that interventions to promote well-being (e.g. targeting environmental influences) can reduce the risk for anxiety or depressive symptoms. For example, in line with the hypothesis of a similar biological mechanism underlying well-being and depression, in adults, a meta-analysis shows that enhancing well-being with positive psychology interventions did decrease depressive symptoms significantly (Sin and Lyubomirsky 2009). In contrast, the lower genetic and environmental correlation of well-being and aggressive behavior suggest more distinct biological and environmental mechanisms underlying both traits. Therefore, a prevention or intervention to increase well-being and decrease aggressive behavior at the same time is most likely not effective.

To conclude, bivariate twin models are powerful models to investigate the causes of covariation between traits. The phenotypic correlations, estimates of bivariate heritability, and genetic and environmental correlations resulting from bivariate twin models provide distinctive information about the covariance and overlap between phenotypes and have different (clinical) implications. It is highly recommended that future behavior genetics studies report both sets of results.

## Supplementary Information

Below is the link to the electronic supplementary material.

Supplementary file1 (PNG 100 KB)

Supplementary file2 (PNG 94 KB)

Supplementary file3 (DOCX 41 KB)

Supplementary file4 (R 10 KB)

Supplementary file5 (R 13 KB)
