# Supplementary figures and images for: Genetic Influences on the Covariance and Genetic Correlations in a Bivariate Twin Model: An Application to Well-Being
Source: Behav Genet. 2021 Feb 13;51(3):191–203. doi: 10.1007/s10519-021-10046-y (PMC8093176; doi:10.1007/s10519-021-10046-y)

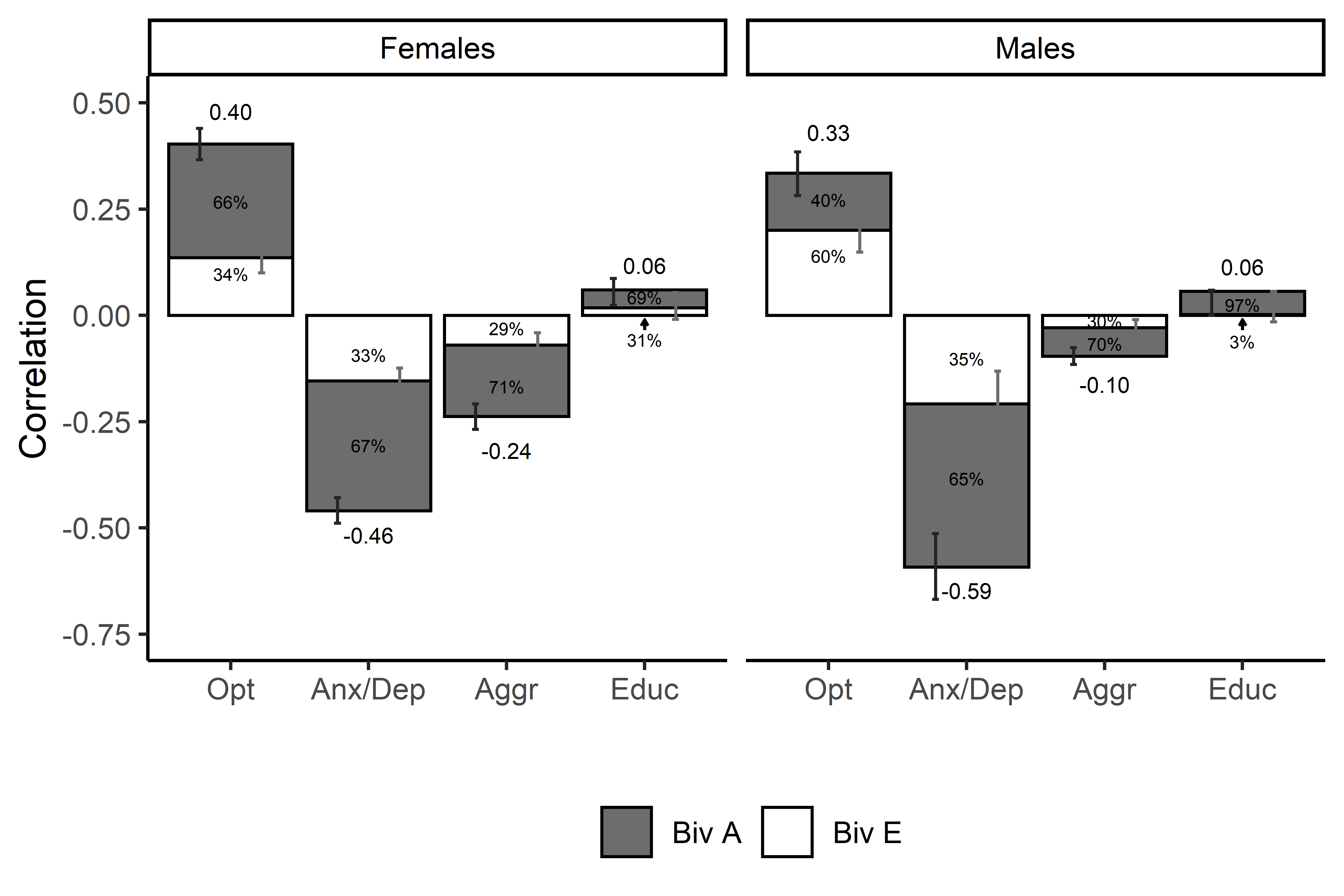

Supplement: Supplementary file 1 — Supplementary file1 (PNG 100 KB) [file 10519_2021_10046_MOESM1_ESM.png]

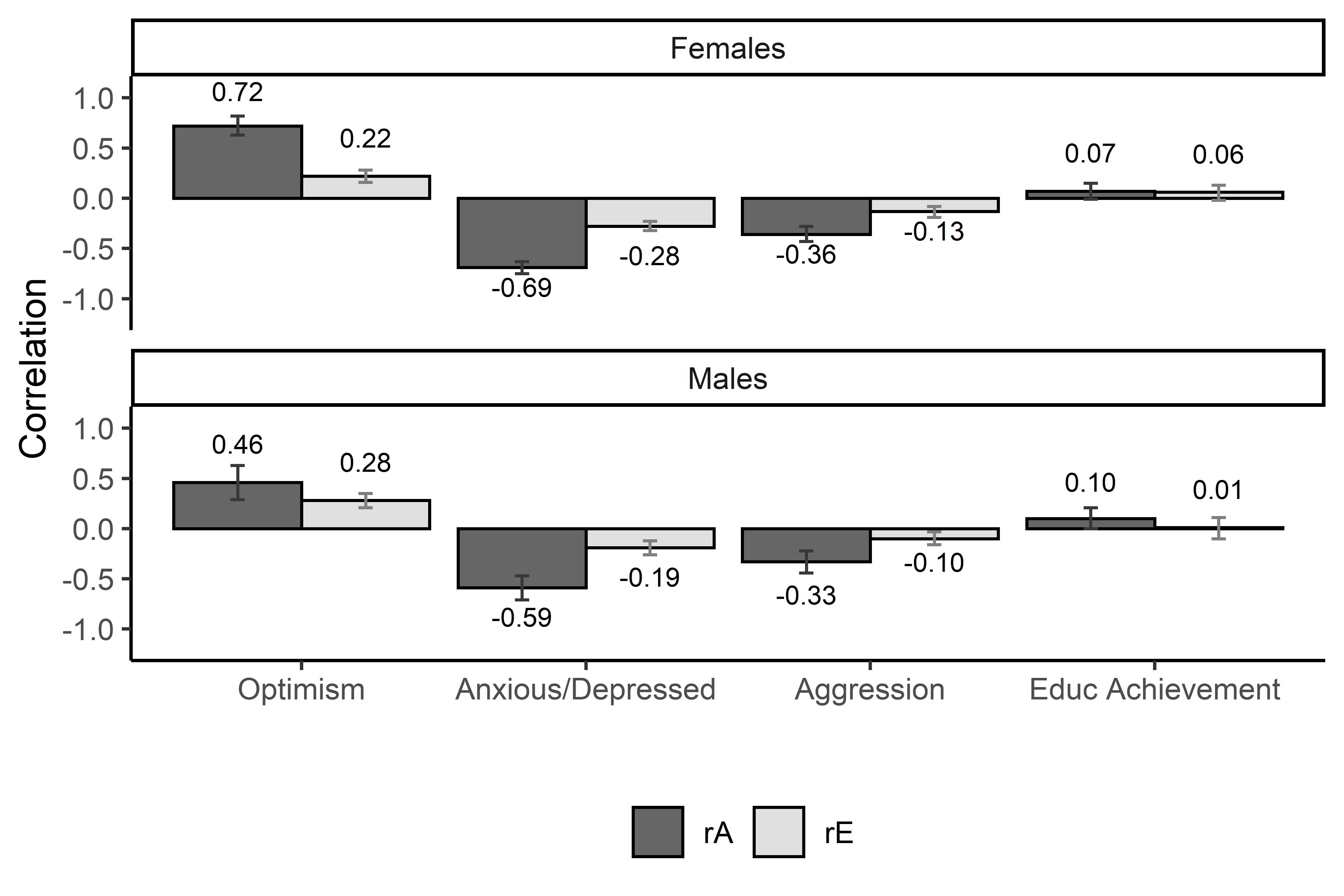

Supplement: Supplementary file 2 — Supplementary file2 (PNG 94 KB) [file 10519_2021_10046_MOESM2_ESM.png]
