# Supplementary material for: Genetic Influences on the Covariance and Genetic Correlations in a Bivariate Twin Model: An Application to Well-Being
Source: Behav Genet. 2021 Feb 13;51(3):191–203. doi: 10.1007/s10519-021-10046-y (PMC8093176; doi:10.1007/s10519-021-10046-y)
Supplement: Supplementary file 3 — Supplementary file3 (DOCX 41 KB) [file 10519_2021_10046_MOESM3_ESM.docx]

**Supplementary Tables**

Table S1.

*The bivariate genetic, shared and non-shared environmental estimates of the four full ACE models.*

*Note*: The NA’s in the CI for the covariance of WB and Educ are a consequence of the small r of .06.

|  | **A** | | **C** | | | **E** | |  |
| --- | --- | --- | --- | --- | --- | --- | --- | --- |
|  | WB | Opt | WB | Opt | | WB | Opt | |
| **Females** |  |  |  |  | |  |  | |
| WB | *.*258  *(.113, .406)* |  | *.*170  *(.041, .293)* |  | | *.*572  *(.528, .618)* |  | |
| Opt | *.*432  *(.112, .761)* | *.*059  *(-.123, .243)* | *.*217  *(-.063, .484)* | *.*225  *(.069, .374)* | | *.*351  *(.252, .453)* | *.*716  *(.658, .777)* | |
| **Males** |  |  |  |  | |  |  | |
| WB | *.*344  *(.151, .539)* |  | *.*001  *(-.165, .160)* |  | | *.*655  *(.595, .720)* |  | |
| Opt | *.*088  *(-.456, .624)* | *.*302  *(.062, .544)* | *.*276  *(-.181, .732)* | -.049  *(-.254, .150)* | | *.*635  *(.475, .804)* | *.*747  *(.674, .825)* | |
|  | **A** |  | **C** |  | | **E** |  | |
|  | WB | Anx-Dep | WB | Anx-Dep | | WB | Anx-Dep | |
| **Females** |  |  |  |  | |  |  | |
| WB | *.*259  *(.114, .407)* |  | *.*168  *(.039, .291)* |  | | *.*573  *(.530, .619)* |  | |
| Anx-Dep | *.*484  *(.236, .740)* | *.*373  *(.218, .533)* | *.*172  *(-.056, .387)* | *.*067  *(-.077, .202)* | | *.*344  *(.276, .416)* | *.*560  *(.517, .606)* | |
| **Males** |  |  |  |  | |  |  | |
| WB | *.*356  *(.164, .550)* |  | -.007  *(-.173, .152)* |  | | *.*651  *(.591, .715)* |  | |
| Anx-Dep | *.*435  *(-.019, .892)* | *.*270  *(.065, .476)* | *.*191  *(-.196, .567)* | *.*111  *(-.065, .277)* | | *.*374  *(.233, .521)* | *.*620  *(.556, .690)* | |
|  | **A** | | | | **C** | **E** | |  |
|  | WB | Aggr | WB | Aggr | | WB | Aggr | |
| **Females** |  |  |  |  | |  |  | |
| WB | *.*277  *(.131, .428)* |  | *.*152  *(.020, .277)* |  | | *.*570  *(.527, .616)* |  | |
| Aggr | *.*514  *(.058, .974)* | *.*530  *(.385, .677)* | *.*177  *(-.223, .566)* | *-.030*  *(-.159, .093)* | | *.*309  *(.177, .446)* | *.*500  *(.458, .546)* | |
| **Males** |  |  |  |  | |  |  | |
| WB | *.*366  *(.172, .560)* |  | *-.017*  *(-.183, .143)* |  | | *.*651  *(.592, .715)* |  | |
| Aggr | *.*080  *(-.661, .788)* | *.*427  *(.251, .605)* | *.*550  *(-.056, 1.18)* | *.*031  *(-.124, .178)* | | *.*370  *(.158, .590)* | *.*543  *(.490, .600)* | |
|  | **A** |  | **C** |  | | **E** |  | |
|  | WB | Educ | WB | Educ | | WB | Educ | |
| **Females** |  |  |  |  | |  |  | |
| WB | *.*277  *(.130, .428)* |  | *.*153  *(.021, .278)* |  | | *.*570  *(.526, .616)* |  | |
| Educ | *.*494  *(NA, NA)* | *.*824  *(.671, .996)* | *.*191  *(NA, NA)* | *-.021*  *(-.190, .129)* | | *.*315  *(-.161, NA)* | *.*196  *(.174, .222)* | |
| **Males** |  |  |  |  | |  |  | |
| WB | *.*364  *(.169, .560)* |  | -.017  *(-.184, .144)* |  | | *.*653  *(.593, .717)* |  | |
| Educ | 2.45  *(NA, NA)* | *.*680  *(.531, .846)* | -1.35  *(NA, NA)* | *.*118  *(-.042, .260)* | | -.097  *(NA, NA)* | *.*201  *(.173, .235)* | |

Table S2.

*The unstandardized genetic, shared and non-shared environmental estimates of the four full ACE models.*

|  | **A** | | **C** | | | **E** | |  |
| --- | --- | --- | --- | --- | --- | --- | --- | --- |
|  | WB | Opt | WB | Opt | | WB | Opt | |
| **Females** |  |  |  |  | |  |  | |
| WB | 7.35  *(NA, NA)* |  | 4.85  *(NA, NA)* |  | | 16.29  *(15.07, 17.64)* |  | |
| Opt | 1.67  *(.42, 2.95)* | *.*19  *(-.40, .78)* | *.*84  *(-.25, 1.90)* | *.*72  *(.22, 1.21)* | | 1.36  *(.96, 1.78)* | 2.29  *(2.10, 2.51)* | |
| **Males** |  |  |  |  | |  |  | |
| WB | 8.40  *(NA, NA)* |  | *.*02  *(NA, NA)* |  | | 16.00  *(14.53, NA)* |  | |
| Opt | 0.25  *(-1.30, NA)* | 0.92  *(.18, 1.67)* | *.*80  *(-.53, 2.10)* | -.15  *(-.78, .46)* | | 1.83  *(1.33, 2.35)* | 2.27  *(2.04, 2.53)* | |
|  | **A** |  | **C** |  | | **E** |  | |
|  | WB | Anx-Dep | WB | Anx-Dep | | WB | Anx-Dep | |
| **Females** |  |  |  |  | |  |  | |
| WB | 7.37  *(NA, NA)* |  | 4.80  *(NA, NA)* |  | | 16.34  *(15.11, 17.70)* |  | |
| Anx-Dep | -4.57  *(NA, NA)* | 5.49  *(NA, NA)* | -1.63  *(NA, NA)* | 0.98  *(NA, NA)* | | -3.25  *(-3.96, -2.58)* | 8.24  *(7.61, 8.93)* | |
| **Males** |  |  |  |  | |  |  | |
| WB | 8.72  *(NA, NA)* |  | -.18  *(NA, NA)* |  | | 15.92  *(14.46, NA)* |  | |
| Anx-Dep | -2.09  *(NA, NA)* | 2.25  *(NA, NA)* | -.92  *(NA, NA)* | 0.92  *(-.56, 2.34)* | | -1.80  *(-2.53, -1.09)* | 5.17  *(4.63, 5.77)* | |
|  | **A** | | | | **C** | **E** | |  |
|  | WB | Aggr | WB | Aggr | | WB | Aggr | |
| **Females** |  |  |  |  | |  |  | |
| WB | 7.93  *(NA, NA)* |  | 4.35  *(NA, NA)* |  | | 16.29  *(15.06, 17.64)* |  | |
| Aggr | -2.28  *(NA, NA)* | 6.42  *(NA, NA)* | -0.79  *(NA, NA)* | -0.37  *(NA, 1.15)* | | -1.38  *(-2.00, -0.77)* | 6.06  *(5.57, 6.61)* | |
| **Males** |  |  |  |  | |  |  | |
| WB | 8.91  *(NA, NA)* |  | -0.41  *(NA, NA)* |  | | 15.87  *(14.41, NA)* |  | |
| Aggr | -0.29  *(NA, NA)* | 6.41  *(NA, NA)* | -2.00  *(NA, NA)* | 0.46  *(NA, NA)* | | -1.34  *(-2.16, -0.55)* | 8.15  *(7.38, 9.01)* | |
|  | **A** |  | **C** |  | | **E** |  | |
|  | WB | Educ | WB | Educ | | WB | Educ | |
| **Females** |  |  |  |  | |  |  | |
| WB | 7.92  *(NA, NA)* |  | 4.36  *(NA, NA)* |  | | 16.27  *(15.05, 17.63)* |  | |
| Educ | 1.37  *(NA, NA)* | 60.95  *(NA, NA)* | *.*53  *(NA, NA)* | *-1.52*  *(NA, NA)* | | *.*88  *(-.37, 2.12)* | 14.50  *(13.01, NA)* | |
| **Males** |  |  |  |  | |  |  | |
| WB | 8.86  *(NA, NA)* |  | -.41  *(NA, NA)* |  | | 15.91  *(15.05, 17.63)* |  | |
| Educ | 5.56  *(NA, NA)* | 44.99  *(NA, NA)* | *-*3.07  *(NA, NA)* | *7.83*  *(NA, NA)* | | *-.*22  *(-.37, 2.12)* | 13.32  *(11.61, NA)* | |

Table S3.

*The genetic, shared environmental, non-shared environmental and phenotypic correlations between well-being and the four other phenotypes of the full ACE models.*

|  | **Optimism** | |  | **Anxious-Depressed symptoms** | |
| --- | --- | --- | --- | --- | --- |
|  | **Females** | **Males** |  | **Females** | **Males** |
| rA | 1.42 *(.489, NA)* | .091 *(-.739, .566)* |  | -.719 *(-1.03, -.429)* | -.473 *(-.976, .027)* |
| rC | .448 *(-.206, .960)* | NA |  | -.749 *(NA, NA)* | NA |
| rE | .222 *(.159, .283)* | .303 *(.226, .376)* |  | -.280 *(-.331, -.227)* | -.198 *(-.272, -.122)* |
| rP | .405 *(.375, .434)* | .334 *(.295, .372)* |  | -.461 *(-.485, -.437)* | -.337 *(-.371, -.301)* |
|  | **Aggression** | |  | **Educational Achievement** | |
|  | **Females** | **Males** |  | **Females** | **Males** |
| rA | -.320 *(-.604, -.039)* | -.039 *(-.370, -.347)* |  | .062 *(-.209, .331)* | .279 *(-.018, .610)* |
| rC | NA | NA |  | NA | NA |
| rE | -.138 *(-.198, -.078)* | -.118 *(-.186, -.049)* |  | .057 *(-.023, .136)* | -.015 *(-.122, .092)* |
| rP | -.239 *(-.269, -.209)* | -.190 *(-.225, -.154)* |  | .060 *(.018, .102)* | .057 *(.007, .106)* |

Note: Biv A= bivariate genetic effects, Biv E= bivariate non-shared environmental effects, rA= additive genetic correlation, rC= shared environmental correlation, rE= non-shared environmental correlation, rP= phenotypic correlation.

Table S4.

*The unstandardized bivariate genetic and non-shared environmental estimates of the four AE models*

|  | **A** |  | **E** |  |
| --- | --- | --- | --- | --- |
|  | WB | Opt | WB | Opt |
| **Females** |  |  |  |  |
| WB | 12.60 (11.18, 14.07) |  | 15.80 (14.68, 17.02) |  |
| Opt | 2.55 (2.13, 2.97) | 0.99 (0.81, 1.18) | 1.30 (0.93, 1.67) | 2.20 (2.03, 2.39) |
| **Males** |  |  |  |  |
| WB | 8.40 (6.81, NA) |  | 16.02 (14.64, NA) |  |
| Opt | 1.16 (0.68, 1.64) | 0.74 (0.51, 0.97) | 1.72 (1.25, 2.21) | 2.29 (2.07, 2.54) |
|  |  |  |  |  |
|  | **A** |  | **E** |  |
|  | WB | Anx-Dep | WB | Anx-Dep |
| **Females** |  |  |  |  |
| WB | 12.59 (11.16, 14.06) |  | 15.85 (14.73, 17.07) |  |
| Anx-Dep | -6.26 (-7.09, -5.46) | 6.51 (5.76, 7.29) | -3.14 (-3.79, -2.52) | 8.17 (7.57, 8.81) |
| **Males** |  |  |  |  |
| WB | 8.47 (6.89, NA) |  | 15.99 (14.61, NA) |  |
| Anx-Dep | -3.12 (-3.87, -2.38) | 3.28 (2.70, 3.87) | -1.69 (-2.36, -1.04) | 5.05 (4.57, 5.60) |
|  |  |  |  |  |
|  | **A** |  | **E** |  |
|  | WB | Aggr | WB | Aggr |
| **Females** |  |  |  |  |
| WB | 12.65 (11.21, 14.14) |  | 15.85 (14.72, 17.08) |  |
| Aggr | -3.13 (-3.86, -2.41) | 6.00 (5.35, 6.67) | -1.30 (-1.88, -0.74) | 6.11 (5.64, 6.63) |
| **Males** |  |  |  |  |
| WB | 8.38 (6.80, NA) |  | 15.97 (14.59, NA) |  |
| Aggr | -2.53 (-3.42, -1.65) | 6.89 (5.95, 7,85) | -1.09 (-1.84, -0.74) | 8.11 (7.39, 8.92) |
|  |  |  |  |  |
|  | **A** |  | **E** |  |
|  | WB | Educ | WB | Educ |
| **Females** |  |  |  |  |
| WB | 12.66 (11.22, 14.15) |  | 15.83 (14.71, 17.06) |  |
| Educ | 1.91 (NA, NA) | 59.52 (NA, NA) | 0.84 (-0.34, 2.03) | 14.53 (13.05, NA) |
| **Males** |  |  |  |  |
| WB | 8.42 (6.84, NA) |  | 15.94 (14.56, NA) |  |
| Educ | 2.21 (NA, NA) | 52.58 (NA, NA) | 0.08 (NA, NA) | 13.06 (11.44, NA) |

Table S5.

*The genetic and non-shared environmental correlations between well-being and the four other traits of the best fitting model.*

|  | **Optimism** | |  | **Anxious-Depressed symptoms** | |
| --- | --- | --- | --- | --- | --- |
|  | **Females** | **Males** |  | **Females** | **Males** |
| rA | .720 *(.626, .816)* | .465 *(.294, .630)* | rA | -.692 *(-.752, -.630)* | -.592 *(-.713, -.471)* |
| rE | .220 *(.161, .277)* | .283 *(.210, .353)* | rE | -.276 *(-.324, -.226)* | -.188 *(-.256, -.117)* |
|  | **Aggression** | |  | **Educational Achievement** | |
|  | **Females** | **Males** |  | **Females** | **Males** |
| rA | -.359 *(-.434, -.282)* | -.333 *(.444, -.223)* | rA | .069 *(-.007, .145)* | .105 *(-.003, .213)* |
| rE | -.132 *(-.188, -.076)* | -.096 *(-.160, -.031)* | rE | .056 *(-.021, .132)* | .005 *(-.096, .107)* |

Note: Biv A= bivariate genetic effects, Biv E= bivariate non-shared environmental effects, rA= additive genetic correlation, rE= non-shared environmental correlation.
